# Accuracy in parameter estimation and simulation approaches for sample-size planning accounting for item effects

**DOI:** 10.3758/s13428-025-02860-7

**Published:** 2026-01-23

**Authors:** Erin M. Buchanan, Mahmoud M. Elsherif, Jason Geller, Chris L. Aberson, Necdet Gurkan, Ettore Ambrosini, Tom Heyman, Maria Montefinese, Wolf Vanpaemel, Krystian Barzykowski, Carlota Batres, Katharina Fellnhofer, Guanxiong Huang, Joseph McFall, Gianni Ribeiro, Jan P. Röer, José L. Ulloa, Timo B. Roettger, K. D. Valentine, Antonino Visalli, Kathleen Schmidt, Martin R. Vasilev, Giada Viviani, Jacob F. Miranda, Savannah C. Lewis

**Affiliations:** 1 Analytics, Harrisburg University of Science and Technology, 326 Market St, Harrisburg, PA 17101 USA; 2 Department of Psychology and Vision Sciences, University of Leicester, Leicester, UK; 3 Department of Psychology and Neuroscience, Boston College, Boston, USA; 4 The Dissertation Coach, Raleigh, NC USA; 5 University of Missouri-St. Louis, St. Louis, MO USA; 6 Department of Neuroscience, University of Padova, Padua, Italy; 7 Methodology and Statistics Unit, Institute of Psychology, Leiden University, Leiden, Netherlands; 8 Department of Developmental and Social Psychology, University of Padova, Padua, Italy; 9 University of Leuven, Leuven, Belgium; 10 Applied Memory Research Laboratory, Institute of Psychology, Faculty of Philosophy, Kraków, Poland; 11 Franklin and Marshall College, Lancaster, PA USA; 12 ETH Zürich, Zürich, Switzerland; 13 Department of Media and Communication, City University of Hong Kong, Kowloon Tong, Hong Kong; 14 Department of Psychology, University of Rochester, Rochester, NY USA; 15 School of Psychology, The University of Queensland, Queensland, Australia; 16 Department of Psychology and Psychotherapy, Witten/Herdecke University, Witten, Germany; 17 Programa de Investigación Asociativa (PIA) en Ciencias Cognitivas, Centro de Investigación en Ciencias Cognitivas (CICC), Facultad de Psicología, Universidad de Talca, Talca, Chile; 18 University of Oslo, Oslo, Norway; 19 Massachusetts General Hospital, Boston, MA USA; 20 IRCCS San Camillo Hospital, Venice, Italy; 21 Ashland University, Ashland, OH USA; 22 Bournemouth University, Poole, UK; 23 California State University East Bay, Hayward, CA USA; 24 University of Alabama, Tuscaloosa, AL USA; 25 Children’s Institute Inc., Los Angeles, CA USA; 26 Harvard Medical School, Boston, MA USA; 27 Research and Innovation Management GmbH, Neumarkt an der Ybbs, Austria

**Keywords:** Accuracy in parameter estimation, Power, Sampling, Simulation, Hypothesis testing

## Abstract

The planning of sample size for research studies often focuses on obtaining a significant result given a specified level of power, significance, and an anticipated effect size. This planning requires prior knowledge of the study design and a statistical analysis to calculate the proposed sample size. However, there may not be one specific testable analysis from which to derive power (Silberzahn et al., *Advances in Methods and Practices in Psychological Science*, *1*(3), 337356, 2018) or a hypothesis to test for the project (e.g., creation of a stimuli database). Modern power and sample size planning suggestions include accuracy in parameter estimation (AIPE, Kelley, *Behavior Research Methods*, *39*(4), 755–766, 2007; Maxell et al., *Annual Review of Psychology*, *59*, 537–563, 2008) and simulation of proposed analyses (Chalmers & Adkins, *The Quantitative Methods for Psychology*, *16*(4), 248–280, 2020). These toolkits offer flexibility in traditional power analyses that focus on the if-this, then-that approach. However, both AIPE and simulation require either a specific parameter (e.g., mean, effect size, etc.) or a statistical test for planning sample size. In this tutorial, we explore how AIPE and simulation approaches can be combined to accommodate studies that may not have a specific hypothesis test or wish to account for the potential of a multiverse of analyses. Specifically, we focus on studies that use multiple items and suggest that sample sizes can be planned to measure those items adequately and precisely, regardless of the statistical test. This tutorial also provides multiple code vignettes and package functionality that researchers can adapt and apply to their own measures.

An inevitable decision in almost any empirical research is deciding on the sample size. Statistical power and power analyses are arguably some of the most important components in planning a research study and its corresponding sample size (Cohen, 1990). However, if reviews of transparency and openness in research publications are any clue, researchers in the social sciences commonly fail to implement proper power analyses as part of their research workflow (Hardwicke et al., 2020, 2022). The replication “crisis” and credibility revolution have shown that published studies in psychology are underpowered (Korbmacher et al., 2023; 2015; 2018. Potential reasons for underpowered studies include questionable research practices (John et al., 2012; but see Fiedler & Schwarz, 2016), weak psychological theories (Proulx & Morey, 2021; Szollosi & Donkin, 2021), testing multiple hypotheses (Maxwell, 2004), and poor intuitions about power (Bakker et al., 2016).

Pre-registration of a study involves outlining the study and hypotheses before data collection begins (Chambers et al., 2014; Nosek & Lakens, 2014; Stewart et al., 2020), and details of a power analysis or limitations on resources are often used to justify the pre-registered sample quota (Pownall et al., 2023; van den Akker et al., 2023a, b). Given the combined issues of publish-or-perish and that most non-significant results do not result in published manuscripts, power analysis may be especially critical for early career researchers to increase the likelihood that they will identify significant effects if they exist (Rosenthal, 1979; Simmons et al., 2011). Justified sample sizes through power analyses may allow for publication of non-significant, yet well-measured effects, along with the smallest effect of interest movement (Anvari & Lakens, 2021), potentially improving the credibility of published work.

A recent review of power analyses found - across behavioral, cognitive, and social science journal articles - researchers did not provide enough information to understand their power analyses and often chose effect sizes that were unjustified (Beribisky et al., 2019). One solution to this power analysis problem is the plethora of tools made available for researchers to make power computations accessible to non-statisticians; however, a solid education in power is necessary to use these tools properly. G*Power is one of the most popular free power software options (Erdfelder et al., 1996; Faul et al., 2007) that provides a simple point and click graphical user interface for power calculations (however, see Brysbaert, 2019). Web-based tools have also sprung up for overall and statistical test specific sample size planning, including https://powerandsamplesize.com, https://jakewestfall.shinyapps.io/pangea/, https://pwrss.shinyapps.io/index/, and https://designingexperiments.com (Anderson et al., 2017). *R*-coding-based packages, such as *pwr* (Champely et al., 2017), *faux* (DeBruine, 2021), *simr* (Green & MacLeod, 2016), *mixedpower* (Kumle & DejanDraschkow, 2020), and *SimDesign* (Chalmers & Adkins, 2020), can be used to examine power and plan sample sizes, usually with simulation. Researchers must be careful using any toolkit, as errors can occur with the over-reliance on software (e.g., it should not be a substitute for critical thinking, Nuijten et al., 2016). Additionally, many tools assume data normality, place an overemphasis on statistical significance, and may rely on simplified assumptions that do not reflect the actual data. Further, the social sciences often ignore robust statistical methods as an option for analysis (Erceg-Hurn & Mirosevich, 2008; Field & Wilcox, 2017), and the implementation of these analyses in power software is somewhat sporadic. Finally, when computing sample-size estimates, it is important to remember that the effect sizes are estimates, not exact calculations guaranteed to produce a specific result (Batterham & Atkinson, 2005). For example, it is hard to estimate all parameters from a study accurately, and if any were incorrect, then the sample size estimate tied to that specific level of power may be incorrect (Albers & Lakens, 2018).

Changes in publication practices and research design have also created new challenges in providing a sample size plan for a research study. While statistics courses often suggest that a specific research design leads to a specific statistical test, meta-science work has shown that given the same data and hypothesis, researchers can come up with multiple ways to analyze the data (Coretta et al., 2023; Silberzahn et al., 2018). Therefore, a single power analysis corresponds only to the specific analysis the researcher expects to implement, and typically, the final expected data ignores any processing pipeline the researcher may use. Analyses may evolve during the research project or be subject to secondary analysis; thus, power and sample size estimation based on one analysis is potentially less useful than previously imagined. Further, research projects often have multiple testable hypotheses, but it is unclear which hypothesis or test should be used to estimate sample size with a power analysis. Last, research investigations may not even have a specific, testable hypothesis, as some projects are intended to curate a large dataset for future reuse (i.e., stimuli database creation, Buchanan et al., 2019).

In light of these analytical (or lack thereof) concerns, we propose a new method to determine a sample size in cases where a more traditional power analysis might be less appropriate or even impossible. This approach combines accuracy in parameter estimation (AIPE, Kelley, 2007; 2008 and data-driven Monte Carlo simulation on pilot data (Rousselet et al., 2022). This method accounts for a potential lack of hypothesis test (or simply no good way to estimate an effect size of interest), and/or an exploratory design with an unknown set of potential hypotheses and analytical choices. Specifically, this manuscript focuses on research designs that use multiple items to measure the phenomena of interest. For example, semantic priming is measured with multiple paired stimuli (Meyer & Schvaneveldt, 1971), which traditionally has been analyzed by creating person or item-level averages to test using an ANOVA (Brysbaert & Stevens, 2018). However, research implementing multilevel models with random effects for the stimuli has demonstrated potential variability in their impact on outcomes; thus, we should be careful not to assume that all items in a research study have the same “effect”.

## Accuracy in parameter estimation

AIPE shifts the focus away from finding a significant *p* value to finding a parameter that is accurately measured. As discussed in Kelley and Maxwell (2003), one can estimate the sample size necessary to obtain precision in the estimation of population parameters. Precision is defined as a researcher-defined, sufficiently narrow, confidence interval in AIPE. For example, researchers may wish to detect a specific mean in a study, *M* = .35. They could then use AIPE to estimate the sample size needed to find a sufficiently narrow window around that mean. Therefore, they could decide that sufficiently narrow could be defined as a width of .30 or .15 on each side of the mean. They would then estimate the number of participants needed to find that level of precision.

Hoekstra et al. (2014) argued that confidence intervals are often misinterpreted (see Miller & Ulrich, 2016 for critique of this claim; see Morey et al., 2016 for original authors’ response), and AIPE procedures are not designed to specify sample size for a hypothesis-driven decision (i.e., the confidence interval does not include a specific value of comparison). Instead, AIPE focuses on estimating the sample size necessary to ensure precise population parameters. Note that any particular confidence interval is not of interest within our procedure, but rather how to define a sufficiently narrow window that a researcher should use for sample size estimation when designing studies with multiple items.

### Monte Carlo simulation

One form of data simulation is data-driven Monte Carlo simulation, which involves using data obtained to simulate similar datasets by drawing from the original data with replacement (Efron, 2000; Rousselet et al., 2022). This type of simulation allows one to calculate parameter estimates, confidence intervals, and to simulate the potential population distribution, shape, and bias. Simulation is often paired with re-creating a data set with a similar structure for testing analyses and hypotheses based on proposed effect sizes or suggested population means. Generally, we would suggest starting with pilot data of a smaller sample size (e.g., 20 to 50, see below for tests to determine the appropriate minimum) to understand the variability in potential items used to represent your phenomenon, especially if they are to be used in a larger study. However, given some background knowledge about the potential items, one could simulate example pilot data to use in a similar manner in our suggested procedure.

Pilot or simulated data would be used to estimate the variability within items and select a sufficiently narrow window for overall item SE for AIPE sufficiently narrow windows. The advantage to this method over simple power estimation from pilot effect sizes is the multiple simulations to average out potential variability, as well as a shift away from traditional NHST to parameter estimation. Simulation would then be used to determine how many participants may be necessary to achieve a dataset wherein as many items as required meet the pre-specified, well-measured criterion.

### Sequential testing

One would set a minimum sample size based on our procedure steps below to ensure an appropriate minimum for precise estimates. After meeting the minimum sample size, researchers could then use sequential testing to estimate their parameter of interest after each participant’s data or at regular intervals during data collection to determine whether they have achieved their expected narrow window around that parameter. A stricter criterion could be defined for a stopping rule (i.e., the criterion that specifies when data collection ends, such as reaching a maximum sample size or achieving a desired level of precision). By defining each of these components, researchers could ensure a feasible minimum sample size, a way to stop data collection when goals have been met, and a maximum sample size rule to ensure an actual end to data collection. The maximum stopping rule could also be defined by resources (e.g., two semesters of data collection), but should nevertheless be included. The advantage of sequential testing lies within research studies that use a random selection of items across participants (i.e., participants do not see all items). Across participants, data can be shifted to items with more uncertainty to increase the precision of estimates for those items. Sequential testing is not a necessary component of our proposed procedure, but we outline how to use the sample size estimates below to set minimum, maximum, and stopping rules. If researchers show all items to participants, they could simply select one of the proposed estimates for their minimum required sample size. Therefore, we propose a method that leverages the ideas behind AIPE, paired with Monte Carlo simulation, to estimate the minimum and maximum proposed sample sizes and stopping rules for studies that use multiple items with expected variability in their estimates. Sequential testing and sequential AIPE methods have a substantial literature of their own (Chow & Chang, 2006; Kelley, 2007; Kelley et al., 2018; Siegmund, 1985; Wald, 1992), and our approach is complementary rather than a replacement for those frameworks.

**Table 1:** Proposed procedure for powering studies with multiple items

Step	Proposed steps	Updated steps
1	Use representative pilot data.	Use representative pilot data.
2	Calculate the standard error of each of the items in the pilot data. Determine the appropriate SE for the stopping rule.	Calculate the standard error of each of the items in the pilot data. Using the 4th decile, determine the cutoff and stopping rule for the standard error of the items.
3	Create simulated samples of your pilot data, starting with at least 20 participants, up to a maximum number of participants.	Create simulated samples of your pilot data, starting with at least 20 participants, up to a maximum number of participants.
4	Calculate the standard error of each of the items in the simulated data. From these scores, calculate the percent of items below the cutoff score from Step 2.	Calculate the standard error of each of the items in the simulated data. From these scores, calculate the percent of items below the cutoff score from Step 2.
5	Determine the sample size at which 80%, 85%, 90%, 95% of items are below the cutoff score.	Determine the sample size at which 80%, 85%, 90%, 95% of items are below the cutoff score. Use the correction formula to adjust your proposed sample size based on pilot data size, power, and percent variability.
6	Report all values. Designate one as the minimum sample size, the cutoff score as the stopping rule for adaptive designs, and the maximum sample size.	Report all values. Designate one as the minimum sample size, the cutoff score as the stopping rule for adaptive designs, and the maximum sample size.

### Proposed method for sample size planning

Building on these ideas, we suggest the following procedure to determine a sample size for each item:

#### Define pilot data and cutoff criterion

 

1. Use pilot data that closely resembles the data you intend to collect. This dataset should contain items that are identical or similar to those that will be implemented in the study. In this procedure, it is important to ensure that the data is representative of a larger population of sampled items that you intend to assess. Generally, pilot data sample sizes will be smaller than the overall intended project (e.g., 20 to 50), as the goal would be to determine how many participants would be necessary to reach a stable standard error for the accurately measured narrow window rule.
2. For each item in the pilot data, calculate the standard error (SE). Select a cutoff SE that defines when items are considered accurately measured. The simulations described in the Data Simulation section will explore what criterion should be used to determine the cutoff SE from the pilot data. Similar concepts appear in classical estimation work where lower bounds of population standard deviations are used as benchmarks (Chattopadhyay & Banerjee, 2021; Mukhopadhyay, 1980).

#### Monte Carlo samples

 

3) Sample, with replacement, from your pilot data using sample sizes starting at a value that you consider the minimal sample size per item, and increase in small units up to a value that you consider the maximum sample size. We will demonstrate example maximum sample sizes based on the data simulation below; however, a practical maximum sample size may be determined by time (e.g., one semester data collection) or resources (e.g., 200 participants worth of funding). As for the minimal sample size, we suggest using 20 as a reasonable value for simulation purposes. For each sample size simulation, calculate the SE for each item. Use multiple simulations (e.g., *n* = 500 to 1000) to avoid issues with random sampling variability.

#### Determine minimum, maximum sample size

 

4) Use the simulated SEs to determine the percentage of items that meet the cutoff score determined in Step 2. Each sample size from Step 3 will have multiple simulations, and therefore, create an average percentage score for each sample size for Step 5.
5) Find the minimum sample size so that 80%, 85%, 90%, and 95% of the items meet the cutoff score and can be considered accurately measured. We recommend these scores to ensure that most items are accurately measured, in a similar vein to the common power-criterion suggestions. Each researcher can determine which of these is their minimum or maximum sample size (e.g., individuals can choose to use 80% as a minimum and 90% as a maximum or use values from Step 3 based on resources).

#### Report results

 

6) Report these values, and designate a minimum sample size, the cutoff/stopping rule criterion, and the maximum sample size. Each researcher should also report if they plan to use an adaptive design, which would stop data collection after meeting the cutoff criterion for each item.

These steps are summarized in Table 1 on the left-hand side. We will first demonstrate the ideas behind the steps using open data (Balota et al., 2007; Brysbaert et al., 2014). This example will reveal a few areas of needed exploration for the steps. Next, we portray simulations for the proposed procedure and find solutions to streamline and improve the sample size estimation procedure. Table 1 shows the results of the simulations and solutions on the right-hand side. Finally, we include additional resources for researchers to use to implement the estimation procedure.

**Table 2:** Sample-size estimates by decile for example study

Deciles	C SE	C 80	C 85	C 90	C 95	L SE	L 80	L 85	L 90	L 95
Decile 1	0.11	115	125	135	150	33.70	170	200	245	345
Decile 2	0.14	65	70	75	85	46.88	90	105	130	180
Decile 3	0.17	50	55	60	65	50.45	80	95	115	160
Decile 4	0.18	45	45	50	55	56.93	60	75	90	125
Decile 5	0.19	40	45	45	50	65.23	50	60	70	95
Decile 6	0.21	35	35	40	45	72.51	40	45	60	80
Decile 7	0.21	35	35	40	45	81.21	30	40	50	65
Decile 8	0.23	30	30	35	40	94.19	25	30	35	50
Decile 9	0.25	25	30	30	35	114.51	20	20	25	35

*Note.* C = Concreteness rating, L = Lexical decision response latencies. Estimates are based on meeting at least the minimum percent of items (e.g., 80%) but may be estimated over that amount (e.g., 82.5%). SE columns represent the standard error value cutoff for each decile, while 80/85/90/95% columns represent the sample size needed to have that percent of items below the SE cutoff. For example, 150 participants are required to ensure at least 95% of concreteness items SE are below the 1st decile SE cutoff, and 345 participants are necessary for the lexical decision SE to be below its 1st decile cutoff

## Example

In this section, we provide an example of the suggested procedure. The first dataset includes concreteness ratings from Brysbaert et al. (2014). Instructions given to participants denoted the difference between concrete (i.e., “refers to something that exists in reality”) and abstract (i.e., “something you cannot experience directly through your senses or actions”) terms. Participants were then asked to rate the concreteness of terms using a 1 (*abstract*) to 5 (*concrete*) scale. This data represents a small-scale dataset (i.e., the range of the scale of the data is small, 4 points) that could be used as pilot data for a study using concrete word ratings. The data is available at https://osf.io/qpmf4/ (see the following for thoughts on analyzing ordinal rating data: Bürkner & Vuorre, 2019; 2023; 2018; 2022.

The second dataset includes a large-scale dataset (i.e., a wide range of possible data values) with response latencies, the English Lexicon Project (ELP, Balota et al., 2007). The ELP consists of lexical decision response latencies for written English words and pseudowords. In a lexical decision task, participants simply select “word” for real words (e.g., *dog*) and “nonword” for pseudowords (e.g., *wug*). The trial-level data are available here: https://elexicon.wustl.edu/. Critically, in each of these datasets, the individual trial-level data for each item is available for simulation and calculation of standard errors. Data that have been summarized could potentially be used, as long as the original standard deviations for each item are present. From the mean and standard deviation for each item, a simulated pilot dataset could be generated for estimating new sample sizes. All code to estimate sample sizes is provided on our OSF page, and this manuscript was created with a *papaja* (Aust et al., 2022) formatted Rmarkdown document.

For this example, imagine a researcher who wants to determine the differences in response latencies for abstract and concrete words. They will select *n* = 40 words from the rating data from Brysbaert et al. (2014) that are split evenly into abstract and concrete ends of the rating scale. In the experiment, each participant will be asked to rate the words for their concreteness and then complete a lexical decision task with these words as the phenomenon of interest. Using both datasets and the procedure outlined above, we can determine the sample size necessary to ensure adequately measured concreteness ratings and response latencies.

*Step 1*. The concreteness ratings data include 27,031 concepts that were rated for their concreteness. We randomly selected *n* = 20 abstract words ($M_{Rating}$ $<= 2$) and *n* = 20 concrete words ($M_{Rating}$ >= 4). In the original study, not every participant rated every word, which created uneven sample sizes for each word. Further, participants were allowed to indicate they did not know a word, and those responses were set to missing data. In our sample of 40 words, the average pilot sample size was 28.52 (*SD* = 1.80), and we will use 29 as our pilot sample size for the concreteness ratings (this information will be used in the follow-up to the simulation study). We first selected the same real words in the ELP data as the concreteness subset selected above, and these data include 27,031 real words. The average pilot sample size for this random sample was 32.67 (*SD* = 0.57), and *n* = 33 will be our pilot size for the lexical decision task.

*Step 2*. Table 2 demonstrates the cutoff scores for deciles of the SEs for the concreteness ratings and lexical decision response latency items. A researcher could potentially pick any of these cutoffs or other percentage options not shown here (e.g., 3.5th decile). We will use simulation to determine the suggestion that best captures the balance of adequately powering our sample and feasibility. This component is explored in the Data simulation section.

*Steps 3–5*. The pilot data were then simulated with replacements, creating samples of 20 to 300 participants per item, increasing in units of 5, for concreteness ratings and lexical decision latencies separately (Step 3). Each of these 57 sample sizes was then repeated 500 times. The SE of each item was calculated for the simulated samples separately for concreteness ratings and lexical decision times (Step 4), and the average percentage of items for each sample size (averaging across the 500 simulations) below each potential cutoff was gathered for each (Step 5). The smallest sample size with at least 80%, 85%, 90%, and 95% of items below the cutoff are reported in Table 2 for each task (Step 5).

*Step 6*. In the last step, the researcher would indicate their smallest sample size, the cutoff SE criterion if they wanted to adaptively test (e.g., examine the SE after each participant and stop data collection if all items reached criteria), and their maximum sample size. As mentioned earlier, the decile for a balanced SE cutoff is unclear and without guidance, a potential set of researcher degrees of freedom could play a role in the chosen cutoff (Simmons et al., 2011). Even though both measurements (ratings and response latencies) appear to converge on similar sample size suggestions for each decile and percent level, the impact of scale size (i.e., concreteness ratings 1–5 versus response latencies in ms 0-3480) and heterogeneity of item standard errors (concrete $SD_{SD}$ = 0.28 and lexical $SD_{SD}$ = 140.83) is not obvious. Last, by selecting the ends of the distribution for our concreteness words, the skew of the distribution may additionally impact our estimates. Each of these will be explored in our simulation.

## Simulation method

In order to evaluate our approach, we used data simulation to create representative pilot datasets of several popular cognitive scales (1–7 measurements, 0–100 percentage measurements, and 0–3000 response latency type scale data). For each of these scales, we also manipulated item heterogeneity by simulating small differences in item variances to large differences in item variances based on original scale size. On each of the simulated datasets, we applied the above proposed method to determine how the procedure would perform and evaluated what criteria should be used for cutoff selection (Step 2). This procedure was performed on distributions in the middle of the scale (i.e., symmetric) and at the ceiling of the scale (i.e., skewed). With this simulation, we will answer several questions:

1. How do pilot data influence sample size suggestions?

A. How does scale size impact sample size estimations? In theory, the size of the scale used should not impact the power estimates; however, larger scales have a potential for more variability in their item standard deviations (see point C).

B. How does distribution skew impact sample size estimations? Skew can potentially decrease item variance heterogeneity (i.e., all items are at ceiling, and therefore, variance between item standard errors is low) or could increase heterogeneity (i.e., some items are skewed, while others are not). Therefore, we expect skew to impact the estimates in the same way as point C.

C. How does heterogeneity impact sample size estimations? Heterogeneity should decrease power (Alexander & DeShon, 1994; Rheinheimer & Penfield, 2001), and thus, increased projected sample sizes should be proposed as heterogeneity of item variances increases.

2) Do the results match what one might expect for traditional power curves? Power curves are asymptotic; that is, they “level off” as sample size increases. Therefore, we expect that our procedure should also demonstrate a leveling off effect as the pilot data sample size increases. For example, if one has a 500-person pilot study, our simulations should suggest a point at which items are likely measured well, which may have happened well before 500.
3) What should the suggested cutoff standard SE be?

### Data simulation

Table 3 presents the variables and information about the simulations as a summary.

**Table 3:** Parameter values for data simulation

Information	Likert	Percent	Milliseconds
Minimum	1.00	0.00	0.00
Maximum	7.00	100.00	3,000.00
$\mu$	4.00	50.00	1,000.00
$Skewed \mu$	6.00	85.00	2,500.00
$\sigma _{\mu }$	0.25	10.00	150.00
$\sigma$	2.00	25.00	400.00
Small $\sigma _{\sigma }$	0.20	4.00	50.00
Medium $\sigma _{\sigma }$	0.40	8.00	100.00
Large $\sigma _{\sigma }$	0.80	16.00	200.00

#### Population

We simulated data for 30 items using the rnorm function assuming a normal distribution. Each item’s population data were simulated with 1000 data points. Items were rounded to the nearest whole number to mimic scales generally collected by researchers. Items were also rounded to their appropriate scale endpoints (i.e., all items below 0 on a 1–7 scale were replaced with 1, etc.).

#### Data scale

The scale of the data was manipulated by creating three sets of scales. The first scale was mimicked after small rating scales (i.e., 1–7 Likert-type style, treated as interval data) using a $\mu$ = 4 with a $\sigma$ = .25 around the mean to create item mean variability. The second scale included a larger potential distribution of scores with a $\mu$ = 50 ($\sigma$ = 10) imitating a 0–100 scale. Last, the final scale included a $\mu$ = 1000 ($\sigma$ = 150) simulating a study that may include response latency data in milliseconds. For the skewed distributions, the item means were set to $\mu$ = 6, 85, and 2500, respectively, with the same $\sigma$ values around the item means. Although there are many potential scales, these three represent a large number of potential variables commonly used in the social sciences. As we suggest, item variances are a key factor in estimating sample sizes, and the scale of the data significantly influences the potential variance. Smaller data ranges (1–7) cannot necessarily have the same variance as larger ranges (0–100).

#### Item Heterogeneity

Next, item heterogeneity was included by manipulating the potential variance for each individual item. For small scales, the variance was set to $\sigma$ = 2 points with a variability of .2, .4, and .8 for low, medium, and high heterogeneity in the variances between items. For the medium scale of the data, the variance was $\sigma$ = 25 with a variance of 4, 8, and 16. Finally, for the large scale of the data, the variance was $\sigma$ = 400 with a variance of 50, 100, and 200 for heterogeneity. These values were based on the proportion of the overall scale and potential variance.

#### Pilot data samples

Each of the populations shown in Table 3 was then sampled as if a researcher were conducting a pilot study. The sample sizes started at 20 participants per item, increasing in units of ten up to 100 participants. Each of these samples would correspond to Step 1 of the proposed method, where a researcher would use pilot data to start their estimation. Therefore, the simulations included 3 scales X 3 heterogeneity values X 2 symmetric/skewed distributions X 9 pilot sample sizes representing a potential Step 1 of our procedure.

#### Assumptions

Our procedure does not assume normality of the data. To illustrate this, we simulated populations with normal, skewed, and bimodal distributions (in special considerations section). What the procedure requires is that item-level scores are sampled from a reasonably stable distribution with finite moments (i.e., mean and variance exist). The method evaluates variability in standard errors rather than relying on strict distributional forms. Thus, while we show results under both normal and non-normal populations, the logic of the procedure is distribution-agnostic provided these basic moment conditions are met.

### Researcher sample simulation

In this section, we simulate what a researcher might do if they follow our suggested application of AIPE to sample size planning based on well-measured items. Assuming that each pilot sample represents a dataset that a researcher has collected (Step 1), the SEs for each item were calculated to mimic the AIPE procedure of finding a sufficiently narrow window. SEs were calculated at each decile of the items up to 90% (i.e., 0% smallest SE, 10% ..., 90% largest SE). The lower deciles would represent a strict criterion for accurate measurement, as many items would need smaller SEs to meet cutoff scores, while the higher deciles would represent less strict criteria for cutoff scores (Step 2).

We then simulated samples of 20 to 2000 increasing in units of 20 to determine what the new sample size suggestion would be (Step 3). We assume that samples over 500 may be considered too large for many researchers who do not work in teams or have participant funds. However, the sample size simulations were estimated over this amount to determine the pattern of suggested sample sizes (i.e., the function between original pilot sample size and projected sample size).

Next, we calculated the percentage of items that fell below the cutoff score, and therefore, would be considered well-measured for each decile by sample (Step 4). From these data, we pinpoint the smallest suggested sample size at which 80%, 85%, 90%, and 95% of the items fall below the cutoff criterion (Step 5). These values were chosen as popular, yet arbitrary, measures of power in which one could determine the minimum suggested sample size (potentially 80% of the items) and the maximum suggested sample size (selected from a higher percentage, such as 90% or 95%).

In order to minimize the potential for random quirks to arise, we simulated the sample selection from the population 100 times and the researcher simulation 100 times for each of those selections. This resulted in 1,620,000 simulations of all combinations of variables (i.e., scale of the data, heterogeneity, data skew, pilot study size, researcher simulation size). The average of these simulations is presented in the results.

## Simulation results

### Pilot data influence on sample size

For each variable, the plot of the pilot sample size, projected sample size (i.e., what the simulation suggested), and power levels are presented below. The large number of variables means we cannot plot them all simultaneously, and therefore, we averaged the results across other variables for each plot. All the datasets can be examined on our OSF page.

**Fig. 1:**
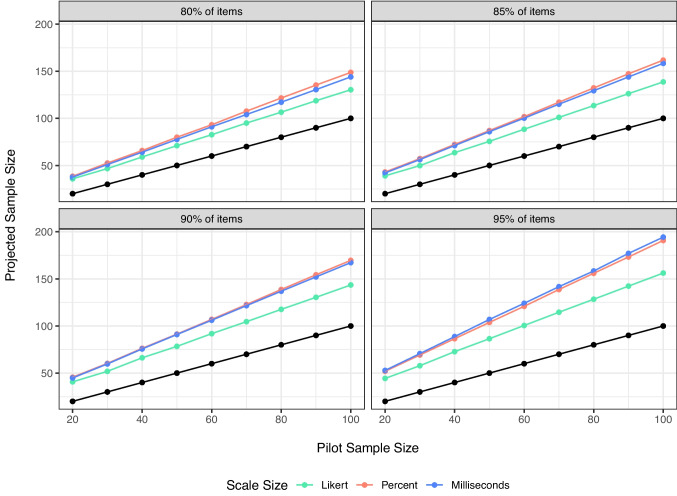
Simulated pilot sample size and final projected sample size to achieve 80%, 85%, 90%, and 95% of items below threshold. These values are averaged over all other variables, including decile. *Black dots* represent original sample size for reference

#### Scale size

Figure 1 demonstrates the influence of scale size on the results separated by potential cutoff decile level. The black dots denote the original sample size for reference. Larger scales have more potential variability, and therefore, we see that percent and millisecond scales project a larger required sample size. This relationship does not appear to be linear with scale size, as percent scales often represent the highest projected sample size. Potentially, this finding is due to the larger proportion of possible variance – the variance of the item standard deviations / total possible variance – was largest for percent scales in this set of simulations ($p_{Percent}$ = .13). This finding may be an interaction with heterogeneity, as the Likert scale had the next highest percent variability in item standard errors ($p_{Likert}$ = .10), followed by milliseconds ($p_{Milliseconds}$ = .06).

**Fig. 2:**
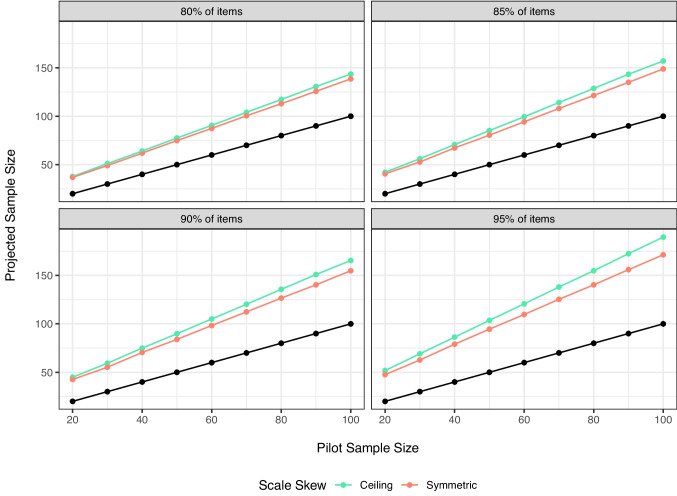
Simulated pilot sample size and final projected sample size to achieve 80%, 85%, 90%, and 95% of items below threshold. In comparison to Fig. 1, this figure shows the projected sample size for ceiling versus symmetric distributions on each scale. All other variables are averaged together, and *black dots* represent original sample size for reference

#### Skew

Figure 2 displays that ceiling distributions, averaged over all other variables, show slightly higher estimates than symmetric distributions. This result is consistent across scale type and heterogeneity, as results indicated that they are often the same or slightly higher for ceiling distributions.

**Fig. 3:**
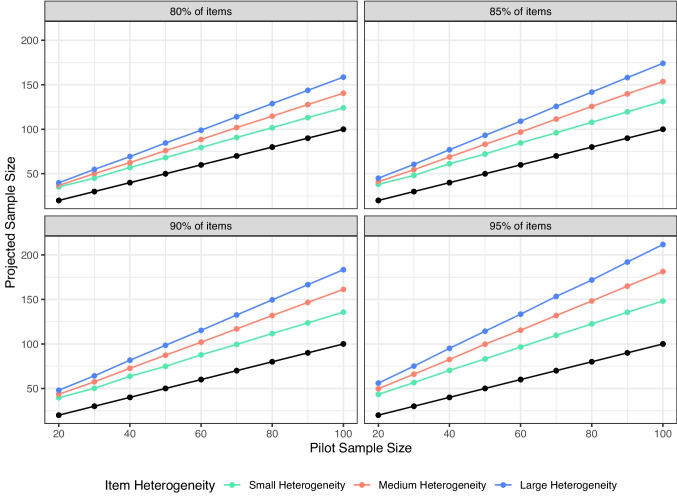
Simulated pilot sample size and final projected sample size to achieve 80%, 85%, 90%, and 95% of items below threshold. In comparison to Figs. 1 and 2, this figure shows projected sample size or differing amounts of heterogeneity on each scale. All other variables are averaged together, and *black dots* represent original sample size for reference

#### Item heterogeneity

Figure 3 displays the results for item heterogeneity for different levels of potential power. In this figure, we found that our suggested procedure does capture the differences in heterogeneity. As heterogeneity increases in item variances, the proposed sample size also increases.

Using a regression model, we predicted proposed sample size using pilot sample size, scale size, proportion variability (i.e., heterogeneity), and data type (symmetric, ceiling). As shown in Table 4, the largest influence on the proposed sample size is the original pilot sample size, followed by the proportion of variance/heterogeneity, and then data and scale sizes.

### Projected sample size sensitivity to pilot sample size

In our second question, we examined whether the suggested procedure was sensitive to the amount of information present in the pilot data. Larger pilot data is more informative, and therefore, we should expect a lower projected sample size. As shown in each figure presented already, we do not find this effect. These simulations from the pilot data would nearly always suggest a larger sample size – mostly in a linear trend increasing with sample sizes. This result comes from the nature of the procedure – if we base our estimates on a SE cutoff, we will almost always need a bit more people for items to meet those goals. This result does not achieve our second goal.

Therefore, we suggest using a correction factor on the simulation procedure to account for the known asymptotic nature of power (i.e., at larger sample sizes, power increases level off). For this function in our simulation study, we combined a correction factor for upward biasing of effect sizes (Hedges’ correction) with the formula for exponential decay calculations. The decay factor was calculated as follows:

$$\begin{aligned} 1 - \sqrt{\frac{N_{Pilot} - min(N_{Simulation})}{N_{Pilot}}}^{log_2(N_{Pilot})} \end{aligned}$$

**Fig. 4:**
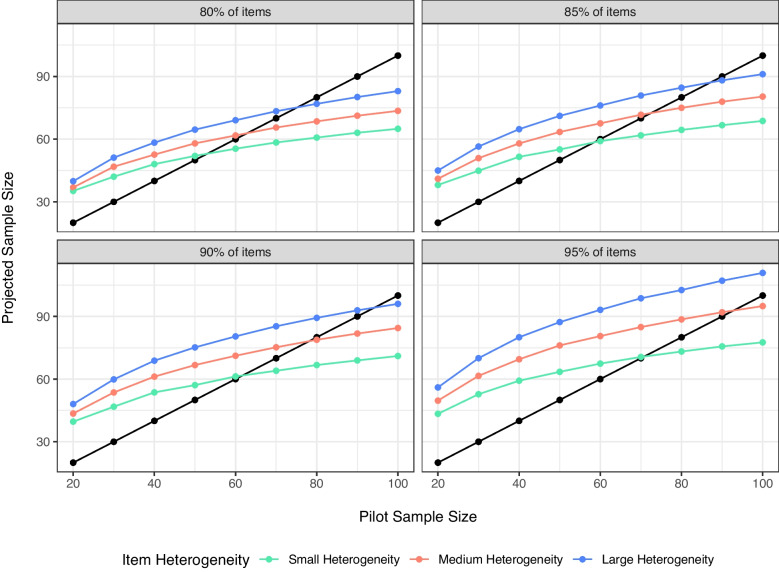
Corrected projected sample sizes for variability and power levels to achieve 80%, 85%, 90%, and 95% of items below threshold. All other variables are averaged together, and *black dots* represent original sample size for reference

$N_{Pilot}$ indicates the sample size of the pilot data minus the minimum simulated sample size to ensure that the smallest sample sizes do not decay (i.e., the formula zeroes out). This value is raised to the power of $log_2$ of the sample size of the pilot data, which decreases the impact of the decay to smaller increments for increasing sample sizes. This value is then multiplied by the projected sample size. As shown in Fig. 4, this correction factor produces the desired quality of maintaining that small pilot studies should *increase* sample size, and that sample size suggestions level off as pilot study data sample size increases.

**Table 4:** Prediction of proposed sample size from simulated variables

Term	Estimate	*SE*	*t*	*p*	$pr^2$
Intercept	-27.30	3.08	-8.87	< .001	.335
Pilot Sample Size	1.51	0.03	54.76	< .001	.951
Scale: Likert v Percent	7.00	1.80	3.89	< .001	.088
Scale: Likert v Millisecond	25.63	1.87	13.74	< .001	.548
Proportion Variability	312.44	19.86	15.73	< .001	.613
Data: Ceiling v Symmetric	-7.16	1.41	-5.08	< .001	.142

**Table 5:** Parameters for all decile cutoff scores

Term	Estimate	*SE*	*t*	*p*
Intercept	111.049	78.248	1.419	.156
Projected sample size	0.429	0.002	185.360	< .001
Pilot sample size	15.434	3.617	4.267	<.001
Log2 projected sample size	-0.718	0.007	-103.787	< .001
Log2 pilot sample size	0.606	0.259	2.343	.019
Log2 power	19.522	0.215	90.693	< .001
Proportion variability	-0.729	0.232	-3.143	.002
Log2 proportion variability	4.655	0.269	17.296	< .001
Power	-39.367	15.640	-2.517	.012

### Corrections for individual researchers

We have shown that this procedure, with a correction factor, can perform as desired. However, within real scenarios, researchers will only have one pilot sample, not the various simulated samples shown above. What should the researcher do to adjust their projected sample size based on their own pilot data simulations?

**Fig. 5:**
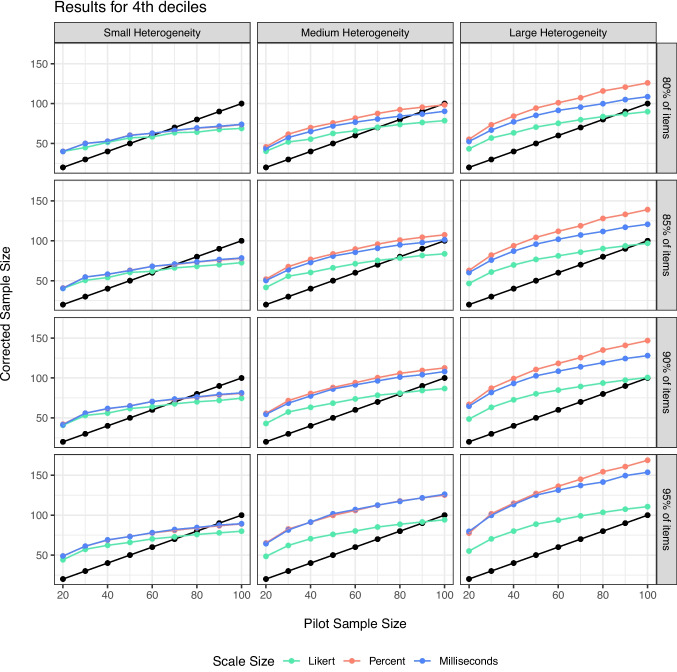
Comparison of the cutoffs for 4th deciles across heterogeneity (*columns*), powering of items (*rows*), and scale size (*color*)

To explore if we could recover the corrected sample size from data a researcher would have, we used regression models to create a formula for researcher correction. The researcher employing our procedure would have the possible following variables from their simulations on their (one) pilot dataset: 1) proposed sample size, 2) pilot sample size, 3) estimate of heterogeneity for the items, 4) and the estimated percent of items below the threshold. Given the non-linear nature of the correction, we added each variable and its non-linear log2 transform to the regression equation, as this function was used to create the correction. The intercept-only model was used as a starting point (i.e., corrected sample ~ 1), and then all eight variables (each variable and their log2 transform) were entered into the regression equation.

As shown in Table 5, all variables were significant predictors of the new sample size. Proposed sample size and original sample size were the largest predictors – unsurprising given the correction formula employed – followed by the percent “power” level and proportion of variance. This formula approximation captures $R^2 = .99$ of the variance in sample size scores and should allow a researcher to estimate based on their own data, $F(8, 4527) = 67,497.54$, $p < .001$. We provide convenience functions in our additional materials to assist researchers in estimating the final corrected sample size.

### Choosing an appropriate cutoff

**Fig. 6:**
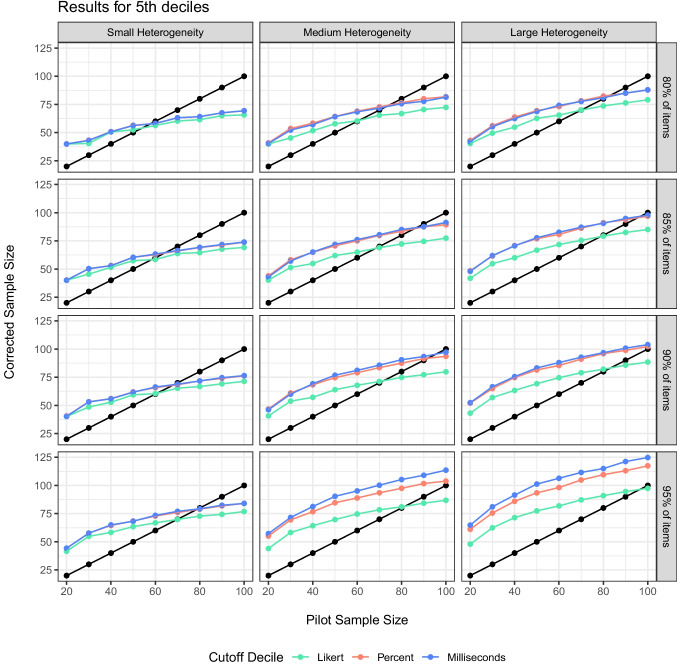
Comparison of the cutoffs for 5th deciles across heterogeneity (*columns*), powering of items (*rows*), and scale size (*color*)

Last, we examined the question of an appropriate SE decile. The minimum and first two deciles are likely too restrictive, providing very large estimates that do not always find a reasonable sample size in proportion to the pilot sample size, scale size, and heterogeneity. If we examine the $R^2$ values for each decile of our regression equation separately, we find that the values are all $R^2$ > .99 with very little differences between them. Figures 5 and 6 illustrate the corrected scores for simulations at the 4th and 5th decile recommended cutoff for item standard errors. For small heterogeneity, differences in deciles are minimal, while larger heterogeneity shows more correction at the 4th decile range, especially for scales with larger potential variance. Therefore, we would suggest the 4th decile to overpower each item for Step 2.

The final formula for 4th decile correction is provided in Table 6. The proportion of variance can be calculated with the following:

$$\begin{aligned} \frac{SD_{Item SD}}{\sqrt{\frac{(Maximum - Minimum)^2}{4}}} \end{aligned}$$

where maximum and minimum are the max and min values found in the scale (or the data, if the scale is unbounded). This formula would be applied in Step 5 of the proposed procedure. While the estimated coefficients could change given variations on our simulation parameters, the general size and pattern of coefficients were consistent, and therefore, we believe this correction equation should work for a variety of use cases. We will now demonstrate the final procedure on the example provided earlier.

**Table 6:** Parameters for 4th decile cutoff scores

Term	Estimate	*SE*	*t*	*p*
Intercept	206.589	128.861	1.603	.109
Projected sample size	0.368	0.005	71.269	< .001
Pilot sample size	-0.770	0.013	-59.393	< .001
Log2 projected sample size	27.541	0.552	49.883	< .001
Log2 pilot sample size	2.583	0.547	4.725	< .001
Log2 power	-66.151	25.760	-2.568	.010
Proportion variability	16.405	6.005	2.732	.006
Log2 proportion variability	-1.367	0.382	-3.577	< .001
Power	1.088	0.426	2.552	.011

## Updated example

The updated proposal steps are in Table 1 on the right-hand side. The main change occurs in Step 2 with a designated cutoff decile, and Step 5 with a correction score. Using the data from the 4th decile in Table 2, we can determine that the stopping rule SE for concreteness ratings would be 0.18, and the stopping rule SE for lexical decision times would be 56.93. For Step 5, we apply our correction formula separately for each one, as they have different variability scores, and these scores are shown in Table 7. Each row was multiplied by row one’s formula, and then these scores are summed for the final corrected sample size. Sample sizes cannot be proportional, so we recommend rounding up to the nearest whole number.

For one additional consideration, we calculated the potential amount of data retention given that participants could indicate they did not know a word ($M_{answered}$ = 0.93, *SD* = 0.11) in the concreteness task or answer a trial incorrectly in the lexical decision task ($M_{correct}$ = 0.80, *SD* = 0.21). In order to account for this data loss, the potential sample sizes were multiplied by $\frac{1}{p_{retained}}$ where the denominator is the proportion retained for each task.

## Additional materials

### Package

We have developed functions to implement the suggested procedure as part of a semantic priming-focused package semanticprimeR. You can install the package from GitHub using: devtools::install_github("SemanticPriming/semanticprimeR"). We detail the functions below with proposed steps in the process.

*Step 1*. Ideally, researchers would have pilot data that represented their proposed data collection. These data should be formatted in long format, wherein each row represents the score from an item by participant, rather than wide format, wherein each column represents an item and each row represents a single participant. The tidyr::pivot_longer() or reshape::melt() functions can be used to reformat wide data. If no pilot data is available, the simulate_population() function can be used with the following arguments (and example numbers, * indicates optional). This function will return a dataframe with the simulated normal values for each item.
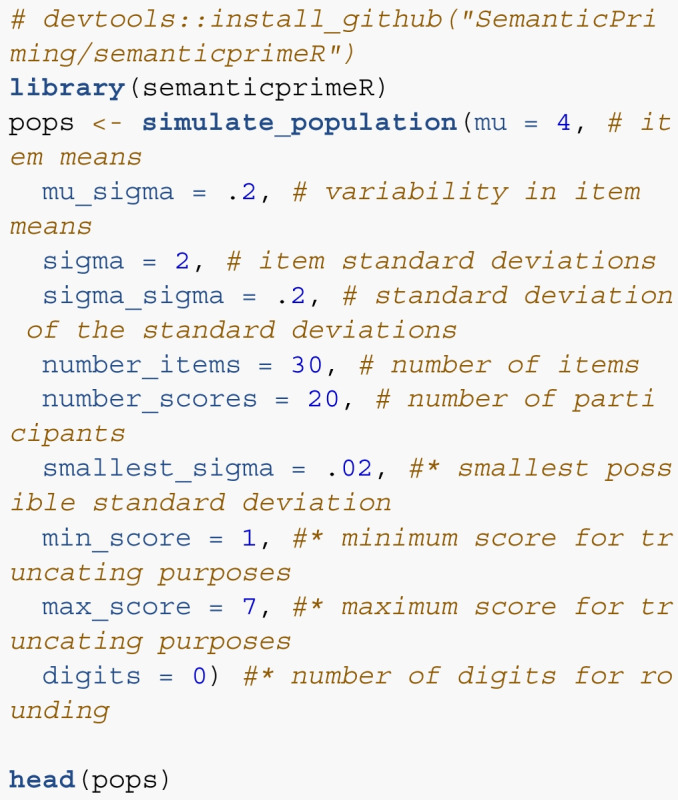

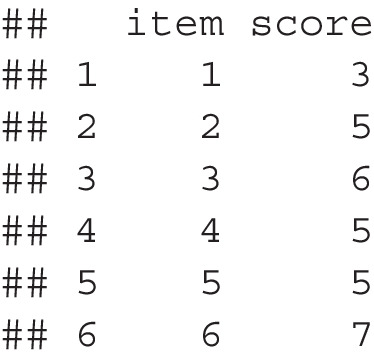

**Table 7:** Applied correction for each proposed sample size

Formula	Intercept	Proj SS	Pilot SS	Log Proj SS	Log Pilot SS	Log Power	Prop Var	Log Prop Var	Power	Loss	Cor SS
Formula	206.59	0.37	-0.77	27.54	2.58	-66.15	16.40	-1.37	1.09	NA	NA
Concrete 80	1.00	45.00	29.00	5.49	4.86	6.32	0.14	-2.82	80.00	39.63	42.56
Concrete 85	1.00	45.00	29.00	5.49	4.86	6.41	0.14	-2.82	85.00	39.29	42.19
Concrete 90	1.00	50.00	29.00	5.64	4.86	6.49	0.14	-2.82	90.00	45.30	48.65
Concrete 95	1.00	55.00	29.00	5.78	4.86	6.57	0.14	-2.82	95.00	51.21	54.99
LDT 80	1.00	60.00	33.00	5.91	5.04	6.32	0.08	-3.60	80.00	54.08	67.68
LDT 85	1.00	75.00	33.00	6.23	5.04	6.41	0.08	-3.60	85.00	68.12	85.25
LDT 90	1.00	90.00	33.00	6.49	5.04	6.49	0.08	-3.60	90.00	80.87	101.20
LDT 95	1.00	125.00	33.00	6.97	5.04	6.57	0.08	-3.60	95.00	107.09	134.00

*Note.* SS = Sample Size, Proj = Projected, Prop = Proportion, Var = Variance, Cor = Corrected

*Step 2*. In step 2, we can use calculate_cutoff() to calculate the standard error of the items, the standard deviation of the standard errors, and the corresponding proportion of variance possible, and the 4th decile cutoff score. The pops dataframe can be used in this function, which has columns named item for the item labels (i.e., 1, 2, 3, 4 or characters can be used), and score for the dependent variable. This function returns a list of values to be used in subsequent steps.
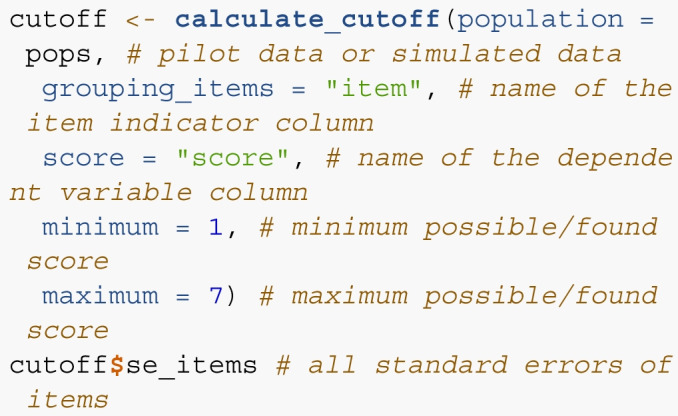

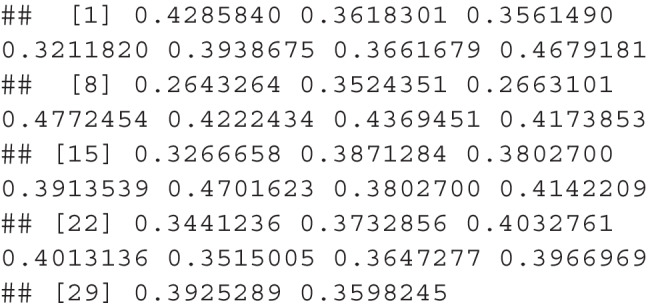

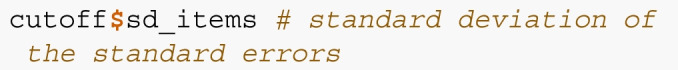

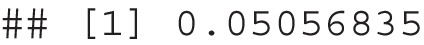

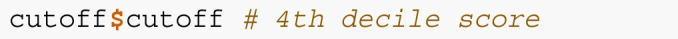

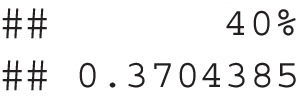

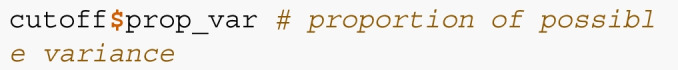

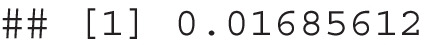


*Step 3*. The simulate_samples() function creates simulated samples from the pilot or simulated population data to estimate the number of participants needed for item standard error to be below the cutoff calculated in Step 2. This function returns a list of samples with sizes that start at the start size, increase by increase, and end with the stop sample size. The population or pilot data will be included in population, and the item column indicator should be included in grouping_items. The nsim argument determines the number of simulations to run.
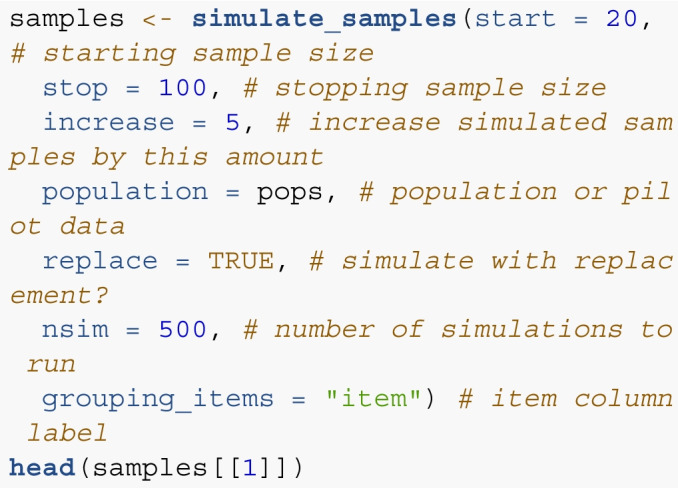

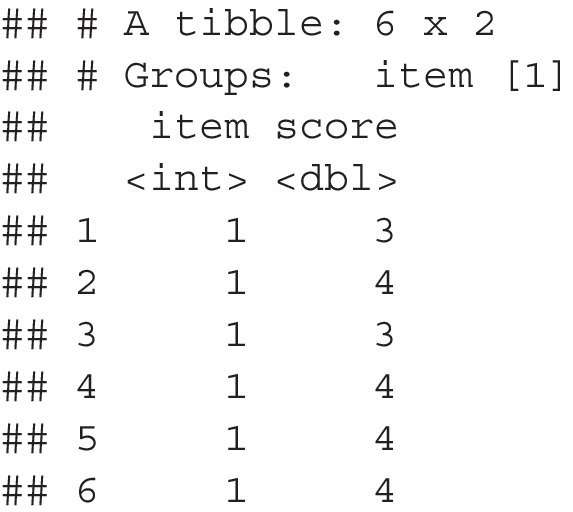


*Step 4 and 5*. The proportion of simulated items across sample sizes below the cutoff score can then be calculated using calculate_proportion(). This function returns a dataframe that includes each sample size and the proportion of items below the cutoff for use in the next function. The samples and cutoff arguments were previously calculated with our functions. The column for item labels and dependent variables are included as grouping_items and score arguments to ensure the right calculations.
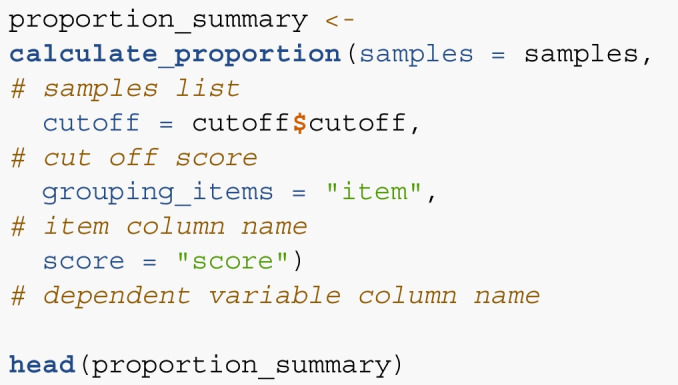

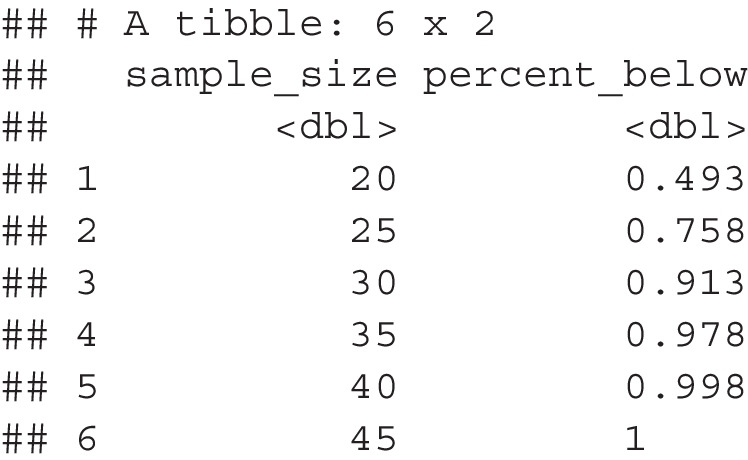


*Step 6*. Last, we use the calculate_correction() function to correct the sample size scores given the proposed correction formula. The proportion_summary from above is used in this function, along with required information about the sample size, proportion of variance from our cutoff calculation, and what power levels should be calculated. Note that the exact percent of items below a cutoff score will be returned if the values in power_levels are not exactly calculated. The final summary presents the smallest sample size, corrected, for each of the potential power levels.
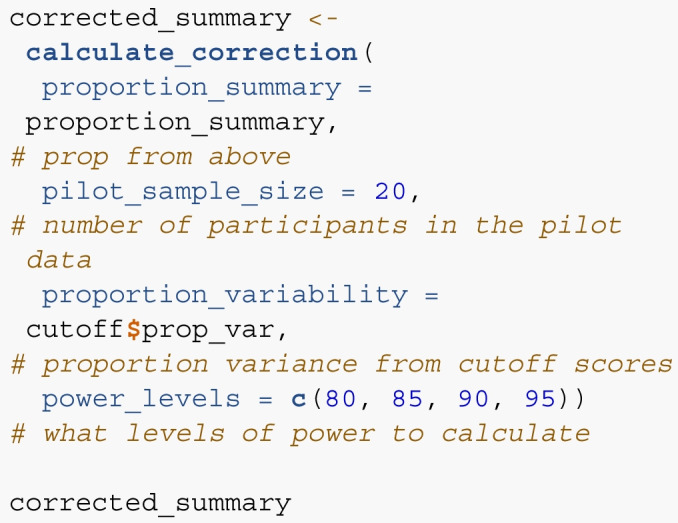

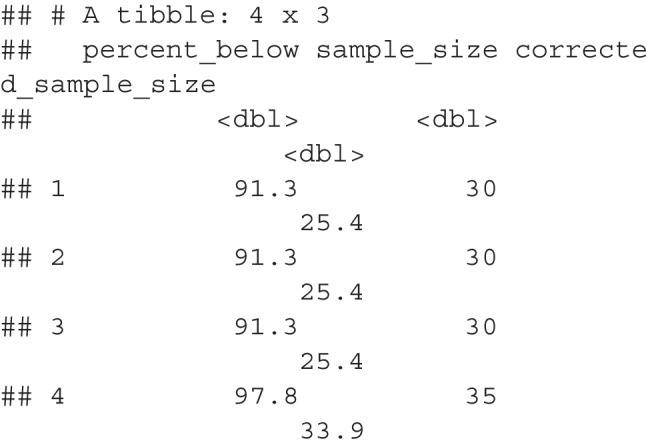


These corrected values represent the final recommended minimum sample sizes for achieving the specified precision thresholds (e.g., 90%, 95%). Researchers can use these numbers directly to determine the target N for their main study, and to define a feasible range of sample sizes. In practice, the corrected N can serve as the minimum sample size, while researchers may also set a maximum sample size based on available resources, and optionally apply sequential monitoring rules to stop data collection early if the desired precision is reached. These corrected estimates translate the pilot-based procedure into concrete targets that researchers can use when planning and monitoring data collection.

### Special considerations

#### Pilot sample size

Smaller pilot samples may be expected to show greater variability than larger samples or the full population. This variability could influence the sample size recommendations generated by our procedure. At the same time, the optimal size of the pilot sample was an open question in our framework. To address these questions, we ran simulations designed to identify the smallest pilot sample size that would minimize variability across researchers while also reducing bias when comparing pilot-derived estimates to population values. In these simulations, 100 researchers each drew a pilot sample from the same target population and applied our procedure. We generated nine populations (the combinations of scale size and heterogeneity described above), each with 30 items (simulate_population). For each population, we varied the pilot sample size across 20, 25, 30, 35, and 40. Each simulated researcher then ran 500 replications, drawing between 20 and 300 participants in increments of 5 (simulate_samples).

We evaluated three statistics from these simulations:

1. Variability of the recommended sample size. For each target level, we calculated the average recommended *N* and the standard deviation of that recommendation. Large standard deviations indicate instability, meaning different researchers could receive very different recommendations from similar pilot studies.
2. Bias in item standard errors. For each item, we compared the estimated *SE* from the sample to the true *SE* from the population. A smaller bias reflects closer agreement between pilot-based estimates and population values. Because larger sample sizes and heterogeneity simulations will naturally show larger SEs in general, we calculated relative error by subtracting the estimated *SE* minus the true *SE* divided by the true *SE*.
3. Cut-off bias. The cut-off was fixed at the 4th decile in the population. For each pilot, we calculated the difference between the pilot-derived cut-off and the population cut-off. Smaller values indicate that pilot-derived thresholds align more closely with the population criterion. Again, we used the relative error by dividing by the true *SE* cut-off score to be able to compare across simulations.

**Fig. 7:**
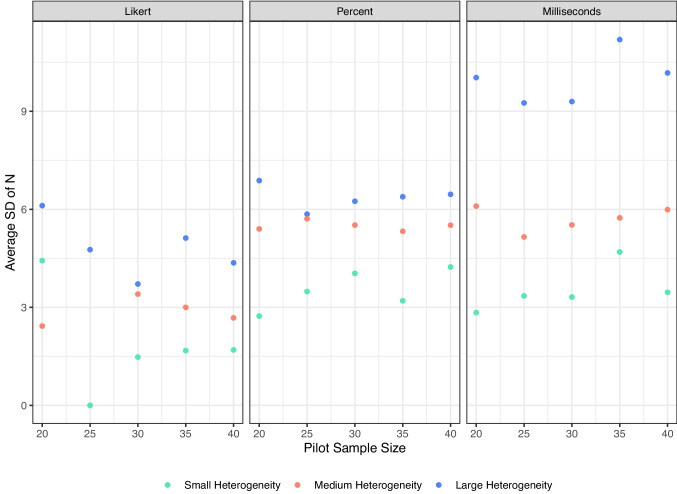
Average standard deviation of suggested sample sizes for small, medium, and large scale sizes and heterogeneity

**Fig. 8:**
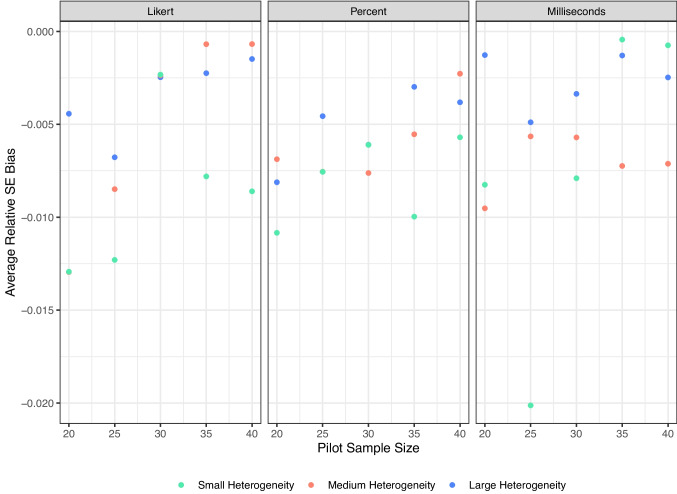
Average relative standard error bias calculated for each simulation of size and heterogeneity combination. Relative error subtracts the pilot sample size SE for each item by the true SE for each item and then divides by the true SE for each item to normalize across scales

**Fig. 9:**
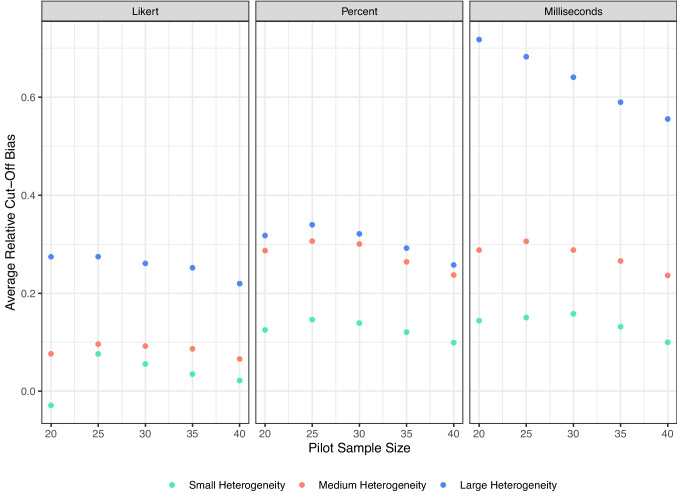
Average relative cut-off score bias for scale size and heterogeneity combinations. Relative cut-off scores indicate the true population 4th decile SE compared to the pilot sample SE cutoff at the 4th decile, divided by the population SE

Results consistently indicated that smaller pilots produced less stable and a bit more biased recommendations. As shown in Fig. 7, the standard deviation of the recommended sample size was higher at pilot sizes of 20 and 25, particularly in large-scale, high-variance populations. This instability means that two researchers running similar small pilots could easily obtain divergent recommended *N*s. By contrast, pilot sizes of 30 or larger substantially reduced this variability, and further increases to 35 or 40 provided little additional gain. Bias measures told a similar story. Figure 8 shows that average item *SE* bias was near zero across most conditions, but small pilots (20–25) tended to produce more negative bias in large-scale populations. At pilot sizes of 30 and above, *SE* bias converged toward zero across all conditions. Likewise, Figure 9 shows that relative cut-off bias was largest when pilot sizes were small, again especially under high-variance conditions, but shrank considerably by pilot size 30 and remained stable thereafter. Accordingly, we propose ~30 participants as a practical lower bound for pilot studies using our procedure: large enough to ensure stability, accuracy, and calibration across a wide range of scale and variance conditions, but small enough to remain efficient.

#### Bimodal data

**Fig. 10:**
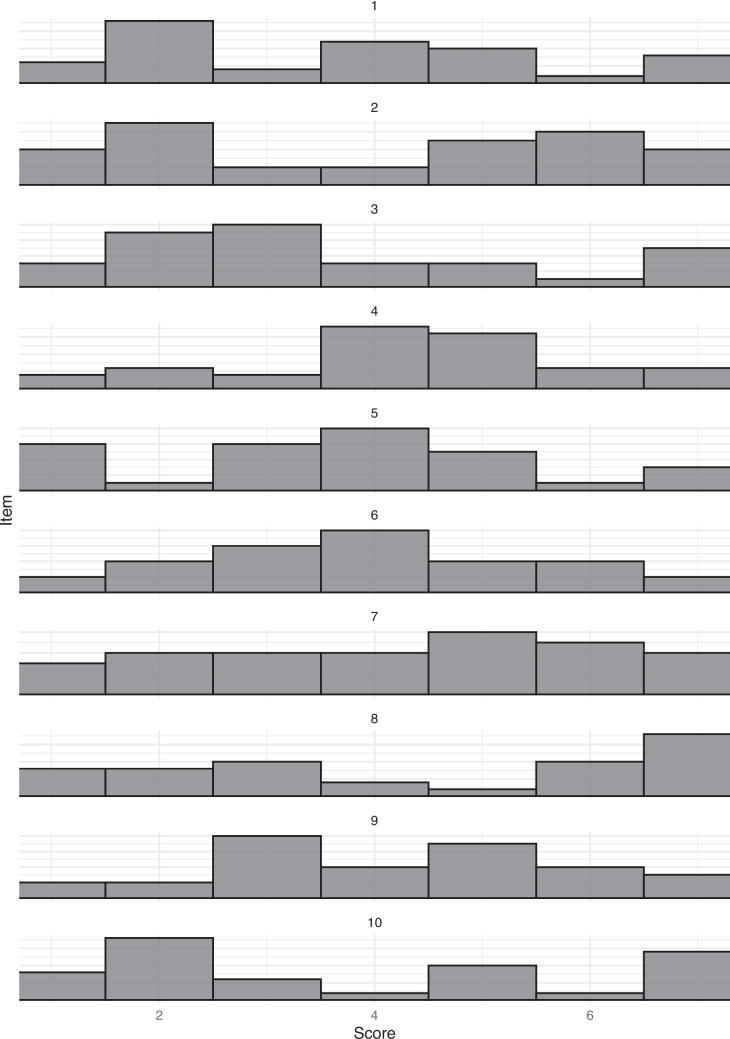
Simulated example of Likert data with half of the items as bimodal distributions

The above simulations assume symmetric population distributions with small, medium, or large heterogeneity or a skewed distribution with the same heterogeneity. However, as shown in Pollock (2018), rating data that appears normally distributed at the aggregate level may in fact reflect the average of two distinct underlying distributions (i.e., a bimodal distribution). In such cases, a pilot sample may misleadingly suggest a single “middle” value that does not represent either true subgroup. To examine our procedure with bimodal distributions, we estimated thirty items for three distributions: symmetric, ceiling, and floor distributions for Likert data with small heterogeneity. Bimodal data was created by selecting half of the ceiling distribution and floor distribution to combine. The number of bimodal items was varied from 0 to 100% increasing by 10% increments for simulated samples. Figure 10 shows an example of ten of the items within a simulation that estimated that half of the Likert items would be bimodal in nature. The researcher procedure described above was then carried out using *semanticprimeR*.

**Fig. 11:**
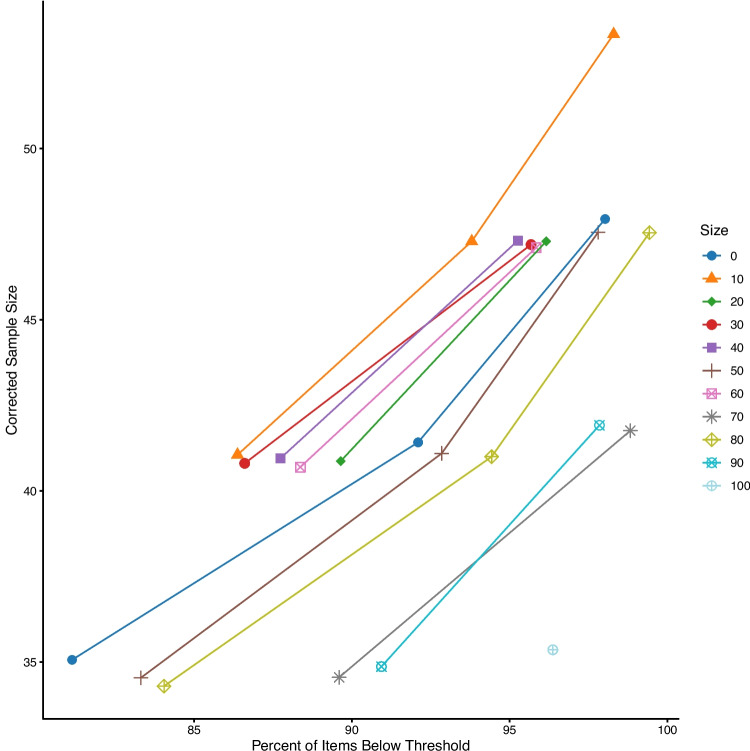
Estimated sample sizes for distributions that vary in the number of bimodal items. Note that the percent below is treated as a continuous factor, and therefore, some estimates are the same (i.e., below 80 and 85% may be both estimated as 88%)

Figure 11 portrays the results of the suggested procedure. The sample with 0 bimodal items demonstrates the same values as above. The proposed sample size increases from 10 to 40% because of the heterogeneity in SE across items (i.e., with 10% of items with a larger SE due to their bimodal nature, the required sample size increases). From 50% to 100% the sample size decreases because the variance of the SEs across items decreases (i.e., at 100%, they are all larger rather than a mix). In theory, these results map onto the conceptual framework – if all items are truly bimodal, we have precisely measured the mean of the bimodal distribution, but this result is likely not the intended result. If researchers expect these distributions (or have representative pilot data), they could examine the data for bimodal items. These items could be estimated separately (i.e., floor effects item 1, ceiling effects item 1) to ensure that both populations of answers are represented in the data.

#### Combining tools

Researchers may often be interested in more than just the precision for individual items. The estimation of power via traditional power calculations for a statistical test or the reliability of items could also be of interest for an appropriate dataset for analysis. We would suggest that researchers combine reliability with precision to collect data that are both reliable and precisely measured. For example, one could use our proposed procedure to calculate the sample size for precision. Separately, the researcher would determine the level of reliability they would like to find in items based on previous research or practical guidelines. The minimum sample size could be set based on estimations for hypothesis testing, the stopping rule based on minimum reliability and desired SE for items, and a maximum sample size based on simulations or practical matters. Each data collection represents a unique scenario in which researchers can combine tools based on their needs to collect precise, reliable, and (traditionally defined) adequately powered data. While our proposal may bring to mind the problems with researcher degrees of freedom (Simmons et al., 2011), transparent practices decisions around sample size planning should be encouraged to limit potential questionable research practices.

### Vignettes

While the example in this manuscript was cognitive linguistics focused, any research using repeated items as a unit of measure could benefit from the proposed newer sampling techniques. Therefore, we provide 12 example vignettes and varied code examples on our OSF page/GitHub site for this manuscript across a range of data types provided by the authors of this manuscript. Examples include psycholinguistics (De Deyne et al., 2008; Heyman et al., 2014; Montefinese et al., 2022), social psychology data (Grahe et al., 2022; Peterson et al., 2022; Ulloa et al., 2014), COVID related data (Montefinese et al., 2021), and cognitive psychology (Barzykowski et al., 2019; Errington et al., 2021; Röer et al., 2013). These can be found on the package tutorial page: https://semanticpriming.github.io/semanticprimeR/.

## Discussion

We proposed a method combining AIPE and Monte Carlo simulation to estimate a minimum and maximum sample size and to define a rule for stopping data collection based on narrow windows on a parameter of interest. In addition, we also demonstrated its practical applications using real-world data. We contend that this procedure is specifically useful for studies with multiple items that intend to use item-level focused analyses; furthermore, the utility of measuring each item well can extend to many analysis choices. By focusing on collecting quality data, we can suggest that the data is useful, regardless of the outcome of any hypothesis test.

One limitation of these methods would be our decision to use datasets with very large numbers of items to simulate what might happen within one study. For example, the English Lexicon Project includes thousands of items, and if we were to simulate for all of those, our results would likely suggest needing thousands of participants for most items to reach the criterion. Additionally, as the number of items increases, you may also see very small estimates for sample size due to the correction factor (as with large numbers of items, you could find many items with standard errors below the 4th decile). Therefore, it would be beneficial to consider only simulating what a participant would reasonably complete in a study. Small numbers of repeated items usually result in larger sample sizes proposed from the original pilot data. This result occurs because the smaller number of items means more samples for nearly all to reach the cutoff criteria. These results are similar to what we might expect for a power analysis using a multilevel model – larger numbers of items tend to decrease the necessary sample size, while smaller numbers of items tend to increase the sample size.

Second, these methods do not ensure the normal interpretation of power, focusing on finding a specific effect for a specific test, $\alpha$, and so on. As discussed in the Introduction, there is not necessarily a one-to-one mapping of hypothesis to analysis; many of the estimations within a traditional power analysis are just that – best approximations for various parameters. These proposed methods and traditional power analysis could be used together to strengthen our understanding of the sample size necessary for both a hypothesis test and a well-tuned estimation. We would advise caution when resampling pilot data (e.g., oversampling beyond the available pilot sample) for power estimation. As Burns et al. (2023) note, resampling introduces estimation bias that increases as simulated sample sizes approach the full pilot size. This bias can occur not only for hypothesis tests but also when estimating descriptive statistics such as means, and it may distort power estimates, particularly in small samples.

Researchers should consider this hybrid approach for AIPE and simulation as a powerful tool for hypothesis testing and parameter estimation. This procedure holds benefits for various research studies, specifically replication studies, that usually prioritize subject sample size but rarely item sample size, in spite of the fact that item sample sizes can contribute to power in multilevel models (Brysbaert & Stevens, 2018; however, see Rouder & Haaf, 2018 for a discussion of the item-sample size trade off). Replicated effects, accumulated through multiple studies and accurate measurement, contribute to robust meta-analyses, enhancing our understanding of the genuine nature of observed effects. This article helps to achieve this goal by encouraging researchers to conduct studies where the power analysis is not based on the size of the effect but on the precise measurement of the stimuli. We argue that this article can be the initial step to apply AIPE, allowing researchers to use item information to provide a more accurate and statistically reliable measure of the effect we aimed to investigate. In conclusion, item power analysis is a tool to avoid the waste of resources while ensuring that items are adequately measured. Well-measured data can enable us to counteract the literature that contains false positives, allowing us to achieve replicable, high-quality science to establish answers to scientific questions with precision and accuracy.

## Data Availability

All data used in this manuscript and vignettes have been cited and can be found on our repository pages (https://github.com/SemanticPriming/stimuli-power).

## Declarations

### Materials

No materials were used.

### Analysis Code

Manuscript repository with code and data: https://osf.io/swmva/ or https://github.com/SemanticPriming/stimuli-power Package repository with vignettes and data: https://github.com/SemanticPriming/semanticprimeR

### Pre-registration

We did not pre-register this study, as it was a simulation study.
